# Navigating the digital era: The impact of digitalization and work-life harmony on well-being among solo self-employed individuals

**DOI:** 10.1371/journal.pone.0350731

**Published:** 2026-06-05

**Authors:** Hyeon Jo, Hyunchul Ahn

**Affiliations:** 1 Headquarters, HJ Institute of Technology and Management, Seoul, Republic of Korea; 2 Kookmin Information Technology Research Institute, Kookmin University, Seoul, Republic of Korea; 3 Graduate School of Business IT, Kookmin University, Seoul, Republic of Korea; International University - Vietnam National University Ho Chi Minh City, VIET NAM

## Abstract

In an era where technological advancements and work-life integration significantly shape the professional landscape, understanding their impact on individual job satisfaction and well-being is crucial, particularly for self-employed business owners. This study explores the effects of digitalization, autonomy, work-life balance, work engagement, and burnout on the job satisfaction and well-being of the self-employed. Using Partial Least Squares Structural Equation Modeling (PLS-SEM) on a sample of 12,703 respondents from the Sixth Korean Working Conditions Survey (2020), this research offers comprehensive insights into the unique challenges faced by this demographic. The findings indicate that digitalization and automation significantly increase technology anxiety. In contrast, leadership autonomy and responsibility enhance job satisfaction but adversely impact well-being. Work-life interference negatively affects job satisfaction and well-being but positively correlates with burnout. Conversely, life-work interference positively influences job satisfaction but negatively impacts work engagement. Both work engagement and job satisfaction positively affect well-being, while burnout shows a negative relationship. Notably, work-life time balance positively influences job satisfaction and well-being, and overtime work has a surprisingly positive effect on these aspects. This research contributes to existing literature by underscoring the distinct experiences of the self-employed in the digital age, laying a groundwork for future research.

## Introduction

Solo self-employed business owners, a growing segment in today’s global market, face unique challenges, particularly amplified by the COVID-19 pandemic (Blackburn et al., 2021). These individuals must navigate significant shifts in market dynamics, customer behaviors, and operational sustainability [1]. The pandemic has underscored the need to explore their job satisfaction and well-being, as these individuals often merge personal and professional lives, making them more vulnerable to external economic and social changes [2,3]. However, despite growing empirical attention, the theoretical mechanisms linking these challenges to well-being outcomes remain insufficiently specified. Accordingly, this study addresses these theoretical gaps by examining how digitally driven demands, boundary dynamics, and psychological responses jointly shape well-being and job satisfaction among solo self-employed individuals.

In today’s fast-paced business environment, shaped by rapid advancements in digitalization and automation, small business owners, particularly those operating solo, encounter complex challenges and opportunities [4]. These technological changes often generate technology anxiety among solo entrepreneurs [5]. Many worry about losing autonomy to automated systems, adapting their skills to a digital landscape, and having their input undervalued. Technology anxiety stems from trying to keep up with new developments and fears of losing control over their businesses. Building on this perspective, this study conceptualizes digitalization/automation as a job demand that induces technology anxiety as a strain response within the Job Demands–Resources (JD-R) health-impairment pathway, ultimately affecting well-being.

Solo self-employed business owners often struggle with work-life balance, particularly work-life interference and life-work interference [6]. Their unique business model blurs the boundaries between personal and professional responsibilities, often leading to work intruding on personal life or vice versa [7]. For example, they might find it difficult to disconnect from work during family events or have trouble focusing on work due to household responsibilities. Building on Boundary Theory, this study differentiates work–life interference and life–work interference as distinct forms of boundary permeability, which are expected to activate different psychological mechanisms and lead to divergent outcomes. It also investigates work-life time balance and the role of overtime, both common among the self-employed, to better understand how these factors impact their job satisfaction and well-being [8].

Work engagement and burnout are critical to understanding the well-being of business owners [9]. High levels of work engagement can enhance job satisfaction and personal fulfillment, leading to greater overall well-being [10,11]. In contrast, burnout, which often results from the high stress and heavy workload of self-employment, can significantly diminish well-being [12]. This study examines how work engagement and burnout interact, exploring their combined impact on the well-being of solo entrepreneurs. Within the JD-R framework, work engagement reflects the motivational pathway driven by internally generated job resources, whereas burnout represents the health-impairment pathway resulting from self-imposed and persistent job demands, both of which are particularly salient in solo self-employment contexts.

Despite the growing interest in entrepreneurial well-being, important theoretical gaps remain. First, prior JD-R research has primarily examined employees in organizational settings, with limited attention to solo self-employed individuals, whose work context lacks clear organizational structures and resource buffering mechanisms [13,14]. As a result, it remains unclear how core JD-R processes—particularly the motivational and health-impairment pathways—operate when job demands and resources are internally generated rather than externally provided. Second, although Boundary Theory has been widely applied to work–family dynamics, it has rarely been integrated with the JD-R model in a process-oriented manner. Existing studies tend to use boundary concepts descriptively, without explaining how different directions of boundary permeability (work-to-life vs. life-to-work) activate distinct psychological mechanisms. Third, the role of technology anxiety has not been theoretically clarified within these frameworks. It is often treated as a direct job demand, despite the possibility that it functions as a secondary psychological strain or cognitive appraisal arising from digitalization-related demands. In particular, this study positions technology anxiety not as a primary job demand, but as a secondary strain response that emerges through the health-impairment pathway. Addressing these gaps, this study integrates JD-R and Boundary Theory to explain how digitally driven demands, boundary dynamics, and psychological responses jointly shape well-being among solo self-employed individuals.

The objective of this study is not only to examine the well-being of solo self-employed individuals but also to advance theoretical understanding by integrating the JD-R model and Boundary Theory in a process-oriented manner. Specifically, this study explicates how distinct job demands and resources activate the motivational and health-impairment pathways, while boundary permeability conditions these processes in self-employment contexts. By doing so, it moves beyond contextual application and contributes to extending the explanatory power of existing theory.

## Theoretical background and research hypotheses

This study is grounded in the JD-R model, which explains how job demands and resources jointly shape well-being and work outcomes [15,16]. Job demands refer to aspects of work that require sustained effort and generate psychological costs, whereas job resources facilitate goal attainment and personal development [17,18]. The JD-R model further distinguishes two underlying processes: the health-impairment pathway, where excessive demands lead to strain and burnout, and the motivational pathway, where resources enhance engagement and job satisfaction [19]. This process-based perspective is particularly relevant for solo self-employed individuals, whose work conditions involve simultaneously self-generated demands and resources [13,20,21].

To complement this perspective, Boundary Theory is incorporated to explain how individuals manage the interface between work and personal life [22]. In solo self-employment contexts, boundaries are often highly permeable, leading to frequent role transitions and increased role conflict [23]. Integrating Boundary Theory with the JD-R model enables a process-oriented explanation of how boundary permeability shapes the experience and impact of job demands and resources.

Within this framework, digitalization/automation is conceptualized as a job demand that increases cognitive load and adaptation pressure, thereby activating the health-impairment pathway. Technology anxiety is treated as a secondary strain response emerging from these demands rather than a primary demand itself [5]. Boundary permeability further intensifies this process by extending exposure to technology-related demands across work and non-work domains.

Leadership autonomy and responsibility are conceptualized as a combined construct reflecting both job resources and demands. Autonomy activates the motivational pathway by enhancing control and self-determination, whereas responsibility triggers the health-impairment pathway due to decision burden and accountability pressures [24,25]. This dual role is particularly pronounced in solo self-employment, where individuals simultaneously experience empowerment and strain.

Work–life interference and life–work interference are conceptualized as boundary-related job demands that operate through distinct mechanisms. Work–life interference reflects work encroachment into personal life, increasing role overload, whereas life–work interference reflects personal demands disrupting work, reducing focus and work-related resources [26,27]. These bidirectional dynamics highlight how boundary permeability differentially activates JD-R pathways.

Finally, work engagement and burnout represent the core outcomes of the motivational and health-impairment pathways, respectively. Work engagement reflects a resource-driven psychological state that enhances well-being, while burnout reflects cumulative strain resulting from excessive demands [28,29]. Together, these constructs capture the dual processes through which work conditions influence well-being in solo self-employment contexts.

**Fig 1:**
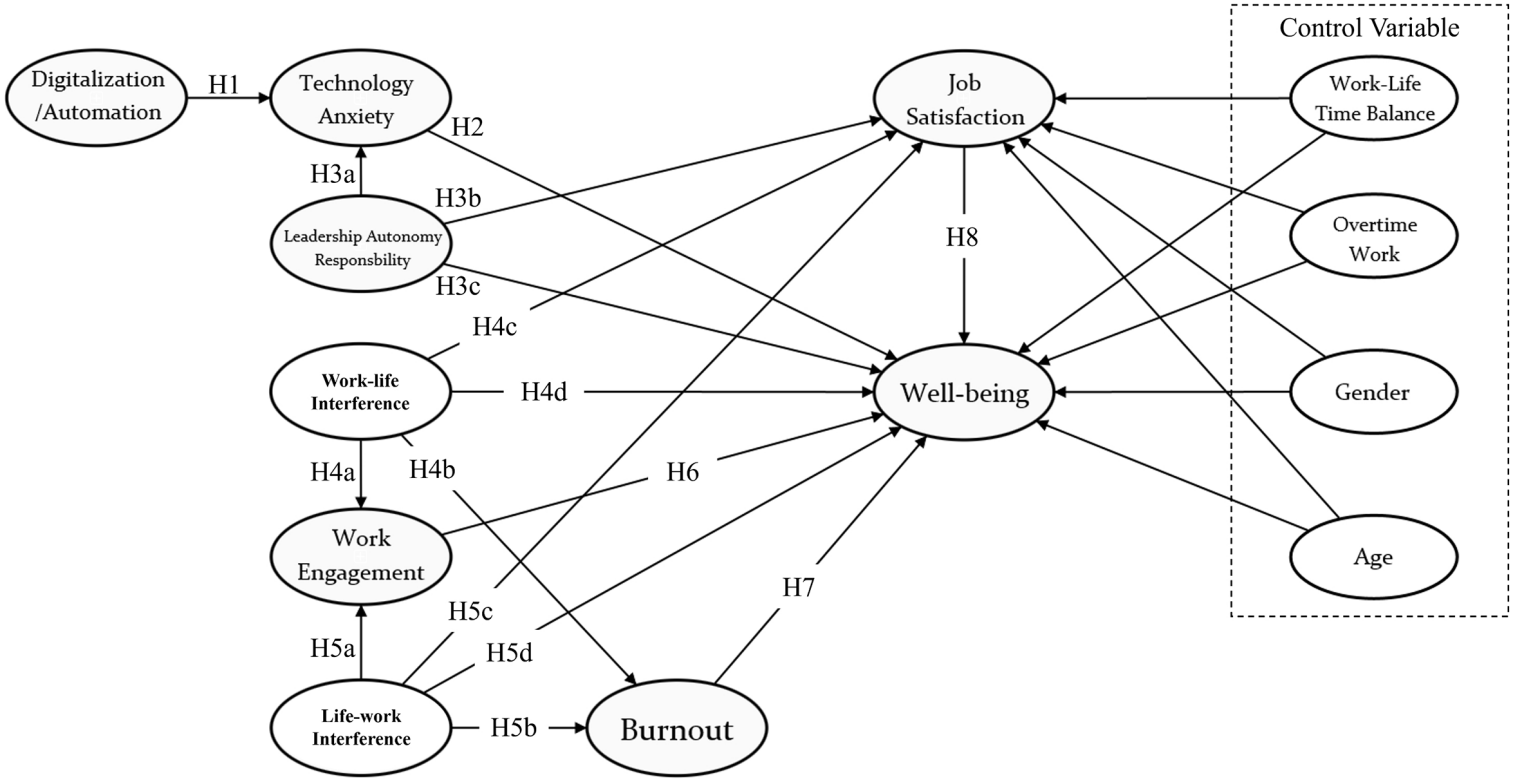
Research framework.

Control variables, including work-life time balance, overtime work, gender, and age, are incorporated as contextual factors that shape the experience of job demands and resources, thereby ensuring a more accurate estimation of the proposed relationships [30–33]. Fig 1 presents the research model.

### Digitalization/automation

Digitalization and automation, integral to modern business practices [34], are particularly impactful for self-employed individuals, often leading to technology anxiety. The transformative nature of these technologies reshapes business operations, posing unique challenges [35]. Research highlights the anxiety among small business owners, particularly those running solo ventures, due to rapid technological changes and the need to adapt [36,37]. From the perspective of the JD-R model, digitalization and automation function as new job demands that require sustained learning and adaptation, thereby increasing cognitive load and psychological strain. These demands are expected to activate the health-impairment pathway of the JD-R model, whereby sustained cognitive load and adaptation pressure deplete psychological resources and manifest as technology-related anxiety. This anxiety is compounded by fears of skill obsolescence and the pressure to stay technologically competitive [38,39]. From a Boundary Theory perspective, continuous digital connectivity blurs the boundaries between work and personal life, making it difficult for solo self-employed individuals to disengage from work-related demands and thereby intensifying psychological strain. This persistent boundary blurring reinforces the development of technology anxiety. Thus, this study suggests the following hypothesis.

H1. Digitalization/Automation positively affects technology anxiety.

### Technology anxiety

Technology anxiety, defined as a state of apprehension or fear related to engaging with technology, has been widely discussed in relation to well-being [40]. This form of anxiety, particularly prevalent among business owners in rapidly digitizing environments, often arises from the need to adapt to new technologies and the fear of being left behind [41]. Prior studies suggest that technology-related stress may negatively influence psychological well-being [42,43]. Within the JD-R framework, however, technology anxiety is more appropriately conceptualized not as a primary job demand, but as a secondary psychological strain or cognitive appraisal emerging from underlying job demands such as digitalization and automation. These demands activate the health-impairment pathway by increasing cognitive load and adaptation pressure, which may translate into anxiety depending on available resources. Accordingly, the impact of technology anxiety on well-being is likely to be indirect and contingent upon mediating mechanisms such as burnout or engagement. From a Boundary Theory perspective, this process is further shaped by boundary permeability. When digital technologies blur work–life boundaries, reduced control over role transitions may intensify anxiety, whereas perceived flexibility may attenuate its negative effects [22,23]. Thus, the following hypothesis is proposed.

H2. Technology anxiety negatively affects well-being.

### Leadership autonomy and responsibility

Leadership autonomy and responsibility, crucial elements in the management of solo businesses, have complex and multi-layered impacts on the psychological state of self-employed individuals [44]. Within the JD-R framework, autonomy and responsibility represent conceptually distinct dimensions, yet they are operationalized as a single construct in this study due to the limitations of the secondary dataset, where both aspects are empirically intertwined. This aggregation reflects the reality of solo self-employment, where decision authority and accountability are experienced simultaneously, although it involves a theoretical trade-off by potentially obscuring distinct JD-R pathways [28]. This operational decision, while empirically justified, may mask the independent effects of autonomy and responsibility, necessitating a more nuanced theoretical interpretation of their combined influence. From a JD-R perspective, autonomy functions as a job resource that activates the motivational pathway, enhancing job satisfaction through increased control, self-determination, and intrinsic motivation [45,46]. In contrast, responsibility reflects a job demand associated with accountability, uncertainty, and performance pressure, thereby activating the health-impairment pathway and increasing psychological strain through sustained cognitive and emotional demands [47]. This strain may manifest as heightened technology anxiety due to the need to independently manage digital adaptation and decision-making under uncertainty [24]. Technology-related uncertainty represents a salient domain in which responsibility-induced strain is cognitively appraised in digitally intensive work environments [48]. From a Boundary Theory perspective, high autonomy and responsibility also increase boundary permeability, as solo self-employed individuals must continuously manage work-related decisions across personal domains. This persistent boundary blurring intensifies role integration and prolongs exposure to job demands, thereby amplifying strain-related outcomes while simultaneously reinforcing motivational outcomes. This dual effect helps explain why autonomy and responsibility simultaneously enhance job satisfaction while increasing strain-related outcomes. Accordingly, the combined construct is expected to exert differential effects through both motivational and health-impairment processes, leading to increased job satisfaction alongside elevated anxiety and reduced well-being. Importantly, these differentiated outcomes are theoretically grounded in the dual-path structure of the JD-R model [28]. Specifically, the resource component of autonomy is expected to influence motivational outcomes such as job satisfaction [49], whereas the demand component of responsibility is expected to influence strain-related outcomes such as technology anxiety and well-being [50]. Therefore, separating these effects into distinct hypotheses is necessary to capture the simultaneous yet opposing mechanisms embedded in solo self-employment contexts. Thus, this study proposes the following hypotheses.

H3a. Leadership autonomy and responsibility positively affect technology anxiety.
H3b. Leadership autonomy and responsibility positively affect job satisfaction.
H3c. Leadership autonomy and responsibility negatively affect well-being.

### Work-life interference

Work-life interference refers to a condition in which work-related demands intrude into personal life domains, disrupting individuals’ ability to maintain clear role boundaries [51]. Within the JD-R framework, work-life interference can be conceptualized as a job demand that requires sustained psychological and emotional effort, thereby activating the health-impairment pathway. Continuous intrusion of work into personal life depletes psychological resources, leading to increased strain outcomes such as burnout and reduced well-being [16]. At the same time, work-life interference may have a more complex relationship with motivational states. In certain contexts, particularly when individuals possess high intrinsic motivation and personal commitment, increased work demands can temporarily intensify focus and involvement in work activities [52]. This suggests that work-life interference may activate a compensatory motivational response, whereby individuals increase their engagement to cope with elevated demands, particularly in contexts characterized by high personal commitment and responsibility [53]. From a Boundary Theory perspective, work-life interference reflects high boundary permeability, where work roles spill over into personal domains. This boundary blurring increases role conflict and reduces opportunities for psychological detachment, thereby intensifying stress and diminishing overall well-being [22]. In solo self-employment contexts, where individuals lack organizational support and role segmentation, such interference is likely to be more pronounced, amplifying its negative consequences [6,54]. This mechanism helps explain why work-life interference predominantly produces strain-related outcomes while only conditionally enhancing engagement. Accordingly, work-life interference is expected to primarily operate through the health-impairment pathway, leading to increased burnout and reduced job satisfaction and well-being. However, under certain conditions, particularly when personal commitment and intrinsic motivation are high, it may also trigger a secondary motivational response, resulting in a short-term increase in work engagement. These differentiated hypotheses reflect the distinct motivational and health-impairment mechanisms through which work-life interference operates within the JD-R framework. Thus, this study proposes the following hypotheses.

H4a. Work-life interference positively affects work engagement.
H4b. Work-life interference positively affects burnout.
H4c. Work-life interference negatively affects job satisfaction.
H4d. Work-life interference negatively affects well-being.

### Life-work interference

Life-work interference refers to a condition in which personal responsibilities intrude into the work domain, disrupting individuals’ ability to maintain focus on professional tasks [51]. Within the JD-R framework, life-work interference can be conceptualized as a job demand that consumes cognitive and emotional resources, thereby activating the health-impairment pathway. Unlike work-life interference, which originates from work demands, life-work interference arises from non-work demands that compete for limited attentional and psychological resources, leading to reduced work engagement and increased strain. Specifically, the intrusion of personal responsibilities into work is likely to reduce work engagement by weakening the motivational pathway, as cognitive distraction and reduced attentional control limit individuals’ capacity for task immersion [27,55]. This continuous role conflict may also contribute to burnout by increasing psychological strain associated with managing competing demands [56]. In addition, such interference can undermine job satisfaction, as difficulties in maintaining work focus may reduce perceived work effectiveness and accomplishment [53,57]. From a Boundary Theory perspective, life-work interference reflects weakened boundary control, where personal roles spill over into the work domain. This reduces individuals’ ability to regulate role transitions and maintain domain separation, thereby increasing role conflict and emotional strain [22]. In the context of solo self-employment, where individuals lack structural boundaries and external regulation, such spillover is more likely to disrupt work processes directly, making its negative impact on work-related outcomes more immediate than work-life interference. This directional difference helps explain why life-work interference more directly undermines motivational outcomes compared to work-life interference. Accordingly, life-work interference is expected to operate primarily through the health-impairment pathway, as it directly disrupts task-related cognitive processes and reduces attentional capacity in work contexts. These differentiated hypotheses reflect the distinct ways in which life-work interference influences motivational and strain-related outcomes within the JD-R framework. Thus, this study proposes the following hypotheses.

H5a. Life-work interference negatively affects work engagement.
H5b. Life-work interference positively affects burnout.
H5c. Life-work interference negatively affects job satisfaction.
H5d. Life-work interference negatively affects well-being.

### Work engagement

Work engagement is characterized by vigor, dedication, and absorption in work [58,59]. When individuals are deeply engaged in their work, they tend to experience higher levels of life satisfaction and psychological well-being [60,61]. Prior research consistently demonstrates that work engagement is positively associated with well-being, as engaged individuals derive meaning, energy, and fulfillment from their work activities [62,63]. Within the JD-R framework, work engagement is not conceptualized as a job resource itself, but as a positive, work-related psychological state that emerges from the availability of job resources and the effective management of job demands. This state reflects the activation of the motivational pathway, whereby sufficient resources (e.g., autonomy, meaningful work) foster engagement, which in turn enhances well-being. Engaged individuals are more resilient to stress and better able to maintain positive psychological functioning even under demanding conditions. Accordingly, work engagement operates as a key mechanism linking job resources to well-being outcomes, rather than as a resource per se. Thus, this study proposes the following hypothesis.

H6. Work engagement positively affects well-being.

### Burnout

Burnout is characterized by emotional exhaustion, depersonalization, and reduced personal accomplishment [64]. The exhaustive nature of burnout significantly erodes an individual’s mental health and life satisfaction [65]. Prior research consistently shows that chronic exposure to stress leads to declines in overall well-being [66,67]. The exhaustion component of burnout is particularly impactful, as it affects not only professional functioning but also personal life, thereby reducing overall life contentment [68]. Within the JD-R framework, burnout represents the core outcome of the health-impairment pathway, where prolonged job demands deplete emotional and cognitive resources over time. When job demands continuously exceed available resources, individuals experience sustained strain, which accumulates into burnout and subsequently undermines well-being. This process highlights how resource depletion serves as a key mechanism linking job demands to negative psychological outcomes. Thus, this study proposes the following hypothesis.

H7. Burnout negatively affects well-being.

### Job satisfaction

Job satisfaction plays a pivotal role in enhancing individual well-being [10]. A positive perception of one’s job contributes significantly to overall life satisfaction and mental health [69,70]. Prior research consistently demonstrates a positive association between job satisfaction and well-being [71,72], suggesting that satisfaction in professional life extends beyond the workplace to influence broader psychological functioning. Within the JD-R framework, job satisfaction reflects the outcome of the motivational pathway, which is activated when job resources such as autonomy, meaningful work, and support foster positive work-related experiences. These resources enhance intrinsic motivation and positive affect, which subsequently translate into higher levels of well-being. In this process, job satisfaction operates as a key mechanism linking job resources to improved psychological outcomes.

H8. Job satisfaction positively affects well-being.

### Control variables

Work-life time balance, overtime work, gender, and age are essential control variables when explaining job satisfaction and well-being due to their strong influence on work-related outcomes. Work-life time balance reflects how effectively individuals manage their personal and professional lives, thereby shaping perceived job demands and overall well-being [30,73]. Overtime work is often associated with increased workload and stress, which may influence burnout and well-being [31,74]. Gender and age also shape workplace experiences, affecting access to resources and perceptions of job satisfaction [32,33,75–77]. Within the JD-R framework, these variables can be interpreted as contextual or background conditions that influence how job demands and resources are experienced, rather than as core theoretical constructs. Therefore, they are included as control variables to isolate the effects of primary predictors. However, given their conceptual proximity to key constructs such as work-life balance and job demands, some overlap may exist, which should be considered when interpreting the results. In addition, due to the use of secondary data, these variables were measured using single-item indicators, which limited the ability to model them as latent constructs. Accordingly, they were operationalized as observed control variables to maintain model parsimony and avoid potential misspecification.

## Research methodology

This study used secondary data from the Sixth Korean Working Conditions Survey (KWCS). The data were fully anonymized before release, and no identifying information was accessible to the researchers. The study was exempt from ethical review, with exemption granted by the Public Institutional Review Board designated by the Ministry of Health and Welfare (Exemption No: P01-202404-01-035). Informed written consent was obtained from all respondents during the original KWCS data collection process, and no additional consent was required for the use of these anonymized secondary data.

The data collection for this study was conducted from 05/10/2020 to 11/04/2021 (approximately 22 weeks). However, due to the spread of COVID-19 and the strengthening of government quarantine measures, field surveys were temporarily suspended for about 45 days, from 13/12/2020 to 26/01/2021.

### Instrument

This study uses data from the 6th Korean Working Conditions Survey (KWCS, 2020–2021), a nationally representative dataset of workers in South Korea. To ensure content validity, survey items from the KWCS were aligned with established constructs in prior literature. Most constructs were measured using Likert-type scales capturing frequency, agreement, or intensity.

Digitalization/automation was measured using three items capturing the extent of technology and automation use at work on a 7-point scale, with higher values indicating greater exposure after reverse coding. Technology anxiety was assessed using three items reflecting concerns about the impact of technological change on work, measured on a 4-point scale and reverse-coded.

Leadership autonomy and responsibility were operationalized using three items capturing decision authority and perceived responsibility, measured on a 5-point scale. Work–life interference and life–work interference were measured using items reflecting bidirectional spillover between work and personal domains, based on established work–family conflict frameworks [26,78], and assessed on a 5-point scale.

Job satisfaction, work engagement, burnout, and well-being were measured using multi-item Likert scales capturing affective and psychological states relevant to work and life outcomes. Control variables, including work-life time balance and overtime work, were measured using single-item Likert scales due to data constraints, as the secondary dataset did not provide sufficient indicators to construct latent variables. Gender and age were included as demographic controls.

All items were reverse-coded where necessary to ensure that higher values consistently reflected higher levels of the constructs. Table 1 presents the measurement items and sources.

**Table 1:** List of constructs and items.

Construct	Item	Measurement	Related work
**Digitalization/automation (7-Likert)**		How many weeks do you spend working in the following ways?	[34,79]
	DTA1	Working with computers, laptops, tablets, and smartphones.	
	DTA2	Working using other internet access devices or equipment to operate machinery.	
	DTA3	Performing tasks that are assigned in an automated manner without human intervention	
**Technology anxiety (4-Likert)**		How worried are you about the following situations that could affect your work in the future due to technological progress and automation:	[80]
	ANX1	A situation in which my right to speak is diminishing.	
	ANX2	A situation where it is difficult to utilize my skills and work skills.	
	ANX3	A situation in which an organization does not have interest in doing things contrary	
**Leadership autonomy and responsibility (5-Likert)**	LAR1	I face a lot of pressure to take responsibility and run my business.	[81]
	LAR2	I make important decisions to run the business.	
	LAR3	I can influence important decisions in the work I do.	
**Work-life interference (5-Likert)**	WLI1	I keep worrying about work even when I’m not working.	[26,51,78,82]
	WLI2	I am so tired after work that I can’t complete the housework I need to do.	
	WLI3	I cannot dedicate as much time to my family as I would like because of work.	
**Life-work interference (5-Likert)**	LWI1	It is difficult to concentrate on work because of housework.	[26,51,78,82]
	LWI2	I can’t spend enough time on my work because of housework.	
**Work engagement (5-Likert)**	EGM1	I feel full of energy when I work.	[58,59]
	EGM2	To be passionate in one’s work.	
	EGM3	Time flies when I work.	
**Burnout (5-Likert)**	BUR1	I feel physically exhausted when I finish work.	[59,64]
	BUR2	I feel mentally exhausted from work.	
**Job satisfaction (5-Likert)**	JSA1	I enjoy running my own business.	[83–85]
	JSA2	Given my efforts and achievements, I am compensated appropriately.	
	JSA3	The organization I work for motivates me to perform my duties to the best of my ability.	
**Well-being (5-Likert)**		In the following questions, please indicate how often you have experienced these feelings in the past two weeks.	[79,86,87]
	WEB1	I am happy and content.	
	WEB2	I feel calm and comfortable.	
	WEB3	I am active and energetic.	
**Work-Life time balance (4-Likert)**	WLT	Are your working hours suitable for family life or social life outside of work?	–
**Overtime work (5-Likert)**	OVW	How often have you worked during free hours outside of your regular working hours in the past year (or since you started your main job if you have been working for less than a year)?	–
**Gender**	GND	What is your gender?	–
**Age**	AGE	How old are you?	–

### Subject and data

This study utilizes data from the 6th Korean Working Conditions Survey (KWCS, 2020–2021), a nationally representative survey of employed individuals aged 15 and above in South Korea. The KWCS adopts a stratified multistage sampling design based on the national census and collects data using a combination of face-to-face interviews and self-administered methods, including Tablet-Assisted Personal Interviewing (TAPI), ensuring high data quality and representativeness across industries and employment types.

The dataset is publicly available and fully anonymized, and the authors did not have access to any personally identifiable information, ensuring compliance with ethical research standards. Given the research focus on entrepreneurial work contexts, the sample was restricted to 12,703 solo self-employed individuals who operate their businesses without permanent employees. This subgroup is particularly suitable for examining work–life dynamics, autonomy, and psychological outcomes in self-managed work environments. This focus enhances the theoretical alignment between the research model and the empirical context.

The large sample size substantially exceeds the minimum requirements for structural equation modeling, thereby enhancing statistical power and the stability of parameter estimates. Moreover, the use of secondary data allows for the examination of real-world working conditions at a population level, increasing the external validity of the findings. Overall, the dataset provides a robust empirical basis for testing the proposed research model. Table 2 outlines the demographic characteristics of the participants in the study.

**Table 2:** Demographic characteristics of the samples.

Variable	Category	Frequency	Percentage
**Gender**	Male	6,234	49.10%
	Female	6,469	50.90%
**Age**	< 40	1,396	11.00%
	40–49	2,150	16.90%
	50–59	3,493	27.50%
	≥ 60	5,664	44.60%
**Education**	≤ Middle school	3,547	27.90%
	High school	5,524	43.50%
	≥ College	3,612	28.40%
**Monthly income (KRW)**	< 2 million	3,557	28.00%
	2–4 million	4,675	36.80%
	≥ 4 million	1,238	9.70%
	Missing/No response	3,233	25.50%

## Analysis and results

The results of this study, conducted using Partial Least Squares Structural Equation Modeling (PLS-SEM) with SmartPLS 4, revealed insightful relationships between various constructs. PLS-SEM was chosen for its ability to handle complex models and large datasets efficiently. It is particularly suited for exploratory research where theoretical underpinnings are still developing, as it allows for the assessment of both measurement and structural models [88]. Additionally, PLS-SEM is preferred for its robustness against deviations from normal distribution, making it ideal for real-world data that often exhibit such non-normality [89]. No author-written or custom code was used in the analysis. All statistical procedures were performed using SmartPLS software, and the results are fully reproducible without the need for additional code. Accordingly, there is no code to share.

### Common method bias

In this study, common method bias was assessed using standard Variance Inflation Factor (VIF) values obtained from the inner model in SmartPLS [90]. Although standard VIF is primarily used to detect multicollinearity among predictors, prior PLS-SEM research suggests that low VIF values can also indicate a reduced likelihood of common method inflation in single-source survey data [88,91]. All standard VIF values were well below the conservative threshold of 3.3, indicating minimal risk of multicollinearity and suggesting that common method bias is unlikely to have substantially distorted the relationships among the constructs. For example, the VIF values for paths such as digitalization/automation → technology anxiety (1.002) and leadership autonomy/responsibility → job satisfaction (1.073) reflect low collinearity, supporting the distinctiveness and independence of the constructs.

### Measurement model

In the measurement model section, the reliability and convergent validity of the constructs were assessed using data from Table 3. The constructs, including digitalization/automation, technology anxiety, leadership responsibility, work-life interference, life-work interference, job satisfaction, work engagement, burnout, and well-being, demonstrated satisfactory levels of reliability and validity. Cronbach’s Alpha and Composite Reliability (CR) values for all constructs were above the recommended threshold of 0.6 and 0.7, respectively, indicating good internal consistency [92]. The Average Variance Extracted (AVE) for each construct exceeded the threshold of 0.5, confirming adequate convergent validity [93].

**Table 3:** Reliability and convergent validity.

Construct	Items	Mean	St. Dev.	Factor Loading	Cronbach’s Alpha	CR	AVE
**Digitalization/automation**	DTA1	2.210	1.788	0.776	0.639	0.806	0.581
	DTA2	1.892	1.524	0.796			
	DTA3	1.367	0.729	0.712			
**Technology anxiety**	ANX1	1.971	0.825	0.901	0.874	0.922	0.798
	ANX2	2.123	0.916	0.895			
	ANX3	2.109	0.862	0.884			
**Leadership autonomy and responsibility**	LAR1	3.372	0.988	0.685	0.608	0.792	0.562
	LAR2	3.854	0.948	0.832			
	LAR3	3.667	1.040	0.724			
**Work-life interference**	WLI1	2.675	1.321	0.729	0.776	0.867	0.687
	WLI2	2.385	1.309	0.920			
	WLI3	2.408	1.479	0.828			
**Life-work interference**	LWI1	1.942	1.276	0.975	0.949	0.975	0.952
	LWI2	1.932	1.276	0.976			
**Work engagement**	EGM1	3.324	0.828	0.899	0.837	0.902	0.754
	EGM2	3.447	0.894	0.899			
	EGM3	3.519	0.868	0.804			
**Burnout**	BUR1	2.699	0.920	0.958	0.907	0.956	0.915
	BUR2	2.591	0.911	0.955			
**Job satisfaction**	JSA1	3.335	0.902	0.763	0.641	0.807	0.582
	JSA2	3.000	0.902	0.788			
	JSA3	2.961	0.783	0.737			
**Well-being**	WEB1	3.725	1.225	0.902	0.882	0.927	0.809
	WEB2	3.969	1.196	0.882			
	WEB3	3.833	1.233	0.914			
**Work-life time balance**	WLT	2.787	0.667	1.000	–	–	–
**Overtime work**	OVW	1.892	0.950	1.000	–	–	–
**Gender**	GND	0.509	0.500	1.000	–	–	–
**Age**	AGE	57.218	13.530	1.000	–	–	–

The Fornell-Larcker criterion, shown in Table 4, and the Heterotrait-Monotrait (HTMT) ratio, from Table 5, were used to establish discriminant validity. The square root of AVE for each construct was higher than its correlations with other constructs, satisfying the Fornell-Larcker criterion [93]. The HTMT values were below the threshold of 0.90, confirming discriminant validity [94]. These results indicate that the constructs in the study are distinct and measure different phenomena, thus ensuring the integrity of the measurement model.

**Table 4:** Fornell-Larcker scale results.

Construct	1	2	3	4	5	6	7	8	9
**1. Digitalization/Automation**	0.762								
**2. Technology anxiety**	0.184	0.894							
**3. Leadership responsibility**	0.043	0.082	0.749						
**4. Work-life interference**	0.041	0.056	0.029	0.829					
**5. Life-work interference**	0.036	0.030	−0.125	0.725	0.975				
**6. Work engagement**	0.134	0.120	0.325	−0.079	−0.116	0.868			
**7. Burnout**	0.080	0.160	−0.028	0.218	0.151	0.054	0.957		
**8. Job satisfaction**	0.137	0.111	0.318	−0.062	−0.045	0.548	−0.041	0.763	
**9. Wellbeing**	0.128	0.071	0.158	−0.091	−0.080	0.581	−0.041	0.437	0.899

Note: The values on the diagonal represent the square root of AVE.

**Table 5:** HTMT matrix.

Construct	1	2	3	4	5	6	7	8	9
**1. Digitalization/automation**									
**2. Technology anxiety**	0.245								
**3. Leadership responsibility**	0.138	0.151							
**4. Work-life interference**	0.076	0.072	0.109						
**5. Life-work interference**	0.047	0.033	0.158	0.832					
**6. Work engagement**	0.176	0.137	0.452	0.086	0.131				
**7. Burnout**	0.107	0.179	0.129	0.248	0.163	0.068			
**8. Job satisfaction**	0.211	0.148	0.497	0.088	0.057	0.741	0.052		
**9. Wellbeing**	0.165	0.080	0.211	0.107	0.088	0.667	0.051	0.578	

### Hypothesis test

In the structural model section of this study, the relationships between various constructs were examined using PLS-SEM, with a bootstrap resampling of 5,000 to ensure robustness. The model explained 37.0% of the variance in well-being, indicating a substantial impact of the included predictors on this outcome. The results are summarized in Table 6.

**Table 6:** Summary of the results.

H	Predictor	Outcome	*β*	*t*	*p*	Result
**H1**	Digitalization/Automation	Technology Anxiety	0.181	21.406	0.000	Supported
**H2**	Technology Anxiety	Well-being	0.003	0.408	0.684	Not Supported
**H3a**	Leadership Autonomy and Responsibility	Technology Anxiety	0.075	7.037	0.000	Supported
**H3b**	Leadership Autonomy and Responsibility	Job Satisfaction	0.310	32.972	0.000	Supported
**H3c**	Leadership Autonomy and Responsibility	Well-being	−0.060	7.225	0.000	Supported
**H4a**	Work-life Interference	Work Engagement	0.012	0.842	0.400	Not Supported
**H4b**	Work-life Interference	Burnout	0.229	17.349	0.000	Supported
**H4c**	Work-life Interference	Job Satisfaction	−0.109	8.630	0.000	Supported
**H4d**	Work-life Interference	Wellbeing	−0.035	3.126	0.002	Supported
**H5a**	Life-work Interference	Work Engagement	−0.125	9.040	0.000	Supported
**H5b**	Life-work Interference	Burnout	−0.015	1.013	0.311	Not Supported
**H5c**	Life-work Interference	Job Satisfaction	0.076	6.175	0.000	Not Supported (Significant)
**H5d**	Life-work Interference	Wellbeing	0.010	0.876	0.381	Not Supported
**H6**	Work Engagement	Wellbeing	0.496	54.837	0.000	Supported
**H7**	Burnout	Wellbeing	−0.058	7.595	0.000	Supported
**H8**	Job Satisfaction	Wellbeing	0.163	17.551	0.000	Supported
**CV**	Work-Life Time Balance	Job Satisfaction	0.163	19.154	0.000	Significant
	Work-Life Time Balance	Well-being	0.031	4.022	0.000	Significant
	Over Time Work	Job Satisfaction	0.018	2.017	0.044	Significant
	Over Time Work	Well-being	0.032	4.729	0.000	Significant
	Gender	Job Satisfaction	0.052	3.253	0.001	Significant
	Gender	Well-being	0.042	2.956	0.003	Significant
	Age	Job Satisfaction	−0.190	23.584	0.000	Significant
	Age	Well-being	−0.054	7.134	0.000	Significant

Note: CV stands for control variable.

The explanatory power of the structural model was assessed using *R²* and *Q²* values. According to [95], *R²* values of 0.25, 0.50, and 0.75 represent weak, moderate, and substantial explanatory power, respectively. As presented in Table 7, technology anxiety (*R²* = 0.039), work engagement (*R²* = 0.014), and burnout (*R²* = 0.048) demonstrate weak explanatory levels, indicating that these outcomes may depend on additional unmeasured personal or contextual factors, such as coping strategies or industry-specific pressures. Job satisfaction (*R²* = 0.166) shows modest explanatory strength, suggesting that leadership autonomy and work–life dynamics explain a meaningful portion of its variance. Well-being yields the highest *R²* (0.370), with a corresponding positive *Q²* value (0.042), indicating acceptable predictive relevance based on the blindfolding procedure [96]. These results collectively suggest that the model predicts well-being more strongly than other endogenous variables.

**Table 7:** Explanatory power (*R²*) and predictive relevance (*Q²*) of endogenous constructs.

Endogenous Construct	*R* ^*2*^	*Q²*
**Technology Anxiety**	0.039	0.039
**Work Engagement**	0.014	0.013
**Burnout**	0.048	0.047
**Job Satisfaction**	0.166	0.164
**Well-being**	0.370	0.042

## Discussion

Digitalization and automation were found to increase technology anxiety, suggesting that technological advancement functions as a salient job demand in solo self-employment contexts. From a JD-R perspective, these demands elevate cognitive load and uncertainty, particularly in the absence of organizational support structures. This finding extends prior research by demonstrating that, for solo entrepreneurs, digitalization is not only an efficiency-enhancing mechanism but also a psychologically taxing condition that intensifies perceived vulnerability to technological change [97].

Contrary to expectations, technology anxiety did not directly reduce well-being. This result suggests that technology anxiety may be more appropriately conceptualized as a secondary psychological strain or cognitive appraisal rather than a primary job demand. Within the JD-R framework, such strain may influence well-being indirectly through mediating mechanisms such as burnout or work engagement. Furthermore, in self-employment contexts, autonomy and flexibility may function as compensatory job resources that buffer the negative effects of technological stress. This finding refines the JD-R model by highlighting the need to distinguish between primary job demands and secondary cognitive-emotional responses in technologically intensive work environments [42,43].

The effects of leadership autonomy and responsibility reveal a theoretically meaningful tension rather than a contradiction. Within the JD-R framework, autonomy operates as a job resource that enhances motivation and satisfaction through increased control, whereas responsibility represents a job demand associated with accountability and performance pressure [28]. The simultaneous positive effect on job satisfaction and negative effect on well-being indicates that these dimensions activate distinct psychological pathways, namely the motivational and health-impairment processes. This tension is particularly pronounced in solo self-employment, where individuals cannot distribute responsibility, thereby amplifying both empowerment and strain. This finding underscores the importance of conceptually separating different leadership components, as aggregating them may obscure distinct JD-R mechanisms.

Work-life interference emerged as a critical job demand that undermines job satisfaction and well-being while increasing burnout. From a Boundary Theory perspective, when work intrudes into personal life, boundary permeability increases and role conflict intensifies, leading to emotional strain and resource depletion [22]. In solo self-employment, where clear boundaries are difficult to maintain, such interference becomes chronic and accumulative, reinforcing the health-impairment pathway of the JD-R model. However, work-life interference did not reduce work engagement. This suggests that engagement, as a motivational state, may be primarily driven by job resources rather than job demands. High autonomy and intrinsic motivation among solo entrepreneurs may sustain engagement even under high interference conditions. This finding refines JD-R assumptions by indicating that not all job demands uniformly diminish engagement, particularly in contexts characterized by high self-determination [19].

Life-work interference demonstrated a contrasting pattern, reflecting the contextual complexity of boundary management. While personal-life intrusion into work reduced engagement, it simultaneously increased job satisfaction, suggesting that such integration may be perceived as flexibility rather than disruption. Boundary Theory explains this duality by emphasizing that boundary crossing can generate both strain and enrichment depending on perceived control [22]. In self-employment contexts, individuals may interpret life-to-work spillover as autonomy-enhancing, thereby increasing satisfaction, even though it disrupts focus and reduces engagement. The absence of significant effects on burnout and well-being further suggests that life-work interference may operate as a low-intensity, context-dependent demand. Within the JD-R framework, burnout is typically driven by sustained and chronic job demands rather than intermittent disruptions [16,29]. Moreover, well-being, as a relatively stable construct, may be more strongly influenced by core work-related factors such as engagement and job satisfaction than by short-term personal interruptions [98,99]. These findings collectively refine existing theoretical assumptions by highlighting the differentiated and context-sensitive nature of boundary dynamics.

The contrasting effects of work-life and life-work interference further emphasize that the direction of boundary crossing is theoretically consequential. Work-to-life interference generates strain by violating personal boundaries, whereas life-to-work interference may introduce flexibility and perceived control. This asymmetry supports Boundary Theory by demonstrating that boundary permeability does not produce uniform outcomes but instead varies depending on which domain is compromised. In the context of solo self-employment, where boundary management is highly individualized, these directional effects become particularly salient, shaping distinct psychological outcomes.

Work engagement showed a strong positive relationship with well-being, reinforcing its role as a key motivational outcome within the JD-R framework. Rather than being a job resource itself, engagement reflects a positive psychological state that emerges from the presence of sufficient job resources and subsequently enhances overall well-being. This finding highlights the importance of fostering resource-rich environments that enable individuals to maintain high levels of energy, dedication, and absorption in their work [99].

Burnout, in contrast, demonstrated a clear negative relationship with well-being, supporting the JD-R model’s health-impairment pathway. Chronic exposure to high job demands without adequate resources leads to emotional exhaustion and resource depletion, ultimately reducing psychological well-being. This finding underscores the critical importance of managing sustained demands in self-employment contexts, where structural support is often limited [66,67].

Job satisfaction positively influenced well-being, confirming its role as a central mechanism linking work experiences to broader life outcomes. Within the JD-R framework, satisfaction reflects the successful activation of the motivational pathway, where job resources translate into positive psychological functioning. This result highlights that fulfilling work experiences extend beyond the workplace and contribute significantly to overall life satisfaction and mental health [71].

Finally, control variables revealed meaningful patterns that warrant further theoretical consideration. The positive effects of overtime work on job satisfaction and well-being suggest that extended working hours may be perceived as voluntary investment rather than coercive demand in self-employment contexts. From a JD-R perspective, this indicates that certain variables traditionally viewed as job demands may operate differently depending on individual autonomy and context. Gender and age differences further indicate that demographic factors shape how individuals experience job demands and resources, influencing both satisfaction and well-being [100–102]. At the same time, the significance of these variables raises the possibility of conceptual overlap with core predictors, particularly in relation to work-life balance and perceived demands. While they were modeled as controls to ensure analytical clarity, their effects suggest that future research may benefit from more explicitly integrating these variables into the theoretical framework.

## Conclusion

### Theoretical contributions

This research’s theoretical contribution offers significant insights into the complex relationship between workplace dynamics and individual well-being and satisfaction.

Building on the JD-R model, this study offers significant contributions by deepening the understanding of how job demands, such as digitalization and automation, affect the psychological state of solo self-employed business owners. Unlike prior research that primarily focused on how technological advancements impact organizational efficiency [103,104], this study uniquely applies the JD-R model to explore the individual psychological repercussions. It demonstrates that digitalization, while enhancing efficiency, also increases technology anxiety, underscoring the dual nature of technological demands. The research adds to the JD-R framework by emphasizing the need for balanced job resources—such as coping mechanisms and supportive technologies—that mitigate the psychological toll of automation and digital transformation. Moreover, including leadership autonomy and responsibility further enriches the JD-R model by showing how decision-making power can simultaneously act as a job resource, boosting satisfaction and as a demand, increasing stress and negatively affecting well-being. This nuanced approach expands the JD-R model’s applicability in self-employment contexts, highlighting the importance of resources that buffer against job demands to preserve well-being.

Additionally, this study contributes to Boundary Theory by illustrating the intricate dynamics between work-life and life-work interference for the self-employed. Boundary Theory traditionally explores how individuals navigate the boundaries between work and personal life [22], but this study goes further by segmenting the dual interference impacts. It reveals that work-life interference, where professional demands spill into personal life, negatively affects well-being and increases burnout. In contrast, life-work interference surprisingly enhances job satisfaction, perhaps due to perceived flexibility in balancing personal tasks within work hours. These findings advance Boundary Theory by revealing that the context of self-employment can alter the traditional understanding of boundary management, showing how the fluidity between work and life can lead to distinct outcomes depending on the direction of the interference. This finding adds depth to the theory by highlighting that, in self-employment, effective boundary management is not only about separation but also about strategically blending roles to optimize well-being and satisfaction.

Thirdly, the significant theoretical contribution is the study’s exploration of the dual effects of leadership autonomy and responsibility. While existing literature, including works by Dess and Lumpkin [81], has often discussed these aspects in the context of organizational leadership and decision-making, this study sheds light on how they distinctly affect self-employed individuals. The research illustrates that while autonomy and responsibility can enhance job satisfaction, they can simultaneously deteriorate well-being due to increased stress. This dichotomy in outcomes is a novel insight, as previous studies have not extensively delved into the simultaneous positive and negative impacts of leadership roles on self-employed individuals.

Finally, this study contributes to the literature by highlighting the paradoxical effect of overtime work on job satisfaction and well-being. Contrary to the traditional view that extended working hours negatively impact workers, the findings here indicate that for self-employed individuals, overtime work can actually lead to increased satisfaction and well-being. This counterintuitive result suggests that the context and nature of work significantly influence how overtime is perceived and experienced, introducing a new dimension to the discussion on work hours and their impact on workers.

### Implications for practitioners

The practical implications of this research are vast and varied, particularly for self-employed business owners, entrepreneurs, the Ministry of SMEs and Startups, and policy-makers.

For self-employed business owners, the findings on digitalization and automation’s impact on technology anxiety are particularly salient. As these individuals often rely heavily on digital tools and automated processes for their businesses, understanding the potential for increased technology anxiety is crucial. It suggests a need for strategies to mitigate stress, such as training programs to enhance digital literacy or using technology that aligns with their skill levels. Additionally, entrepreneurs should be encouraged to seek peer support or professional guidance to adapt to technological advancements effectively, ensuring they can leverage these tools without overwhelming stress.

The dual effects of leadership autonomy and responsibility on job satisfaction and well-being offer critical insights for individual entrepreneurs. While autonomy in decision-making can be empowering and lead to higher job satisfaction, it also comes with increased stress that can impact well-being. Entrepreneurs should be aware of this trade-off and consider implementing stress management practices, like setting clear boundaries between work and personal time or delegating tasks when possible. This balance is key to maintaining not only a successful business but also a healthy personal life.

For the Ministry of SMEs and Startups and policy-makers, the study’s findings on work-life and life-work interference have significant implications. Policies that support flexible work arrangements could be beneficial, allowing business owners more control over their schedules. This flexibility can lead to increased job satisfaction without necessarily compromising work engagement. Additionally, initiatives that promote mental health awareness and provide resources for stress management can help mitigate the negative impacts of work-life interference. Programs that encourage a healthy work-life balance could be instrumental in enhancing both job satisfaction and well-being among entrepreneurs.

Finally, the paradoxical effect of overtime work on job satisfaction and well-being among self-employed individuals offers an interesting insight for policy-making. It suggests that rather than strictly regulating working hours, a more nuanced approach that considers the nature of the work and the individual’s attitude towards overtime may be more effective. Policy-makers could focus on ensuring that overtime work, when undertaken, is by choice and not compulsion, and that it is adequately compensated. Support systems, such as access to childcare services for entrepreneurs who might need to work extended hours, could also be beneficial.

### Limitation and future research

A unique limitation of this study is its focus on solo self-employed business owners, which, while offering detailed insights, may not fully represent the broader workforce, particularly individuals in larger organizations or different cultural contexts. Future research could extend this inquiry by incorporating more diverse business structures and cultural settings to examine how these factors influence the relationships between work dynamics and individual well-being. In addition, longitudinal designs would provide a deeper understanding of how work–life balance, technology anxiety, and leadership responsibilities evolve over time, beyond the cross-sectional snapshot offered here. Exploring the role of emerging technologies, such as artificial intelligence (AI) and machine learning, may also yield forward-looking insights for both scholars and practitioners. A further limitation concerns the aggregation of autonomy and responsibility into a single construct due to constraints of the secondary dataset. This operationalization may obscure their distinct roles as job resources and job demands within the JD-R framework. Future research should disentangle these dimensions to examine their independent and interactive effects. Finally, the use of single-item and two-item measures for certain constructs may limit the ability to fully capture their multidimensional nature and introduce measurement error, thereby affecting reliability compared to multi-item scales. An additional limitation concerns the conceptual treatment of certain variables, such as work-life time balance and overtime work, as control variables. Although these variables were included to isolate the effects of primary predictors, their significant relationships with job satisfaction and well-being suggest potential conceptual overlap with key constructs within the JD-R framework. This raises the possibility that some control variables may function as contextual job demands or resources rather than purely exogenous controls. Future research should more explicitly integrate these variables into the theoretical model, rather than treating them solely as control variables, to better capture their role in shaping work-related outcomes.
